# Supplementary figures and images for: Poorer subjective mental health among girls: Artefact or real? Examining whether interpretations of what shapes mental health vary by sex
Source: PLoS One. 2023 Dec 27;18(12):e0295704. doi: 10.1371/journal.pone.0295704 (PMC10752563; doi:10.1371/journal.pone.0295704)

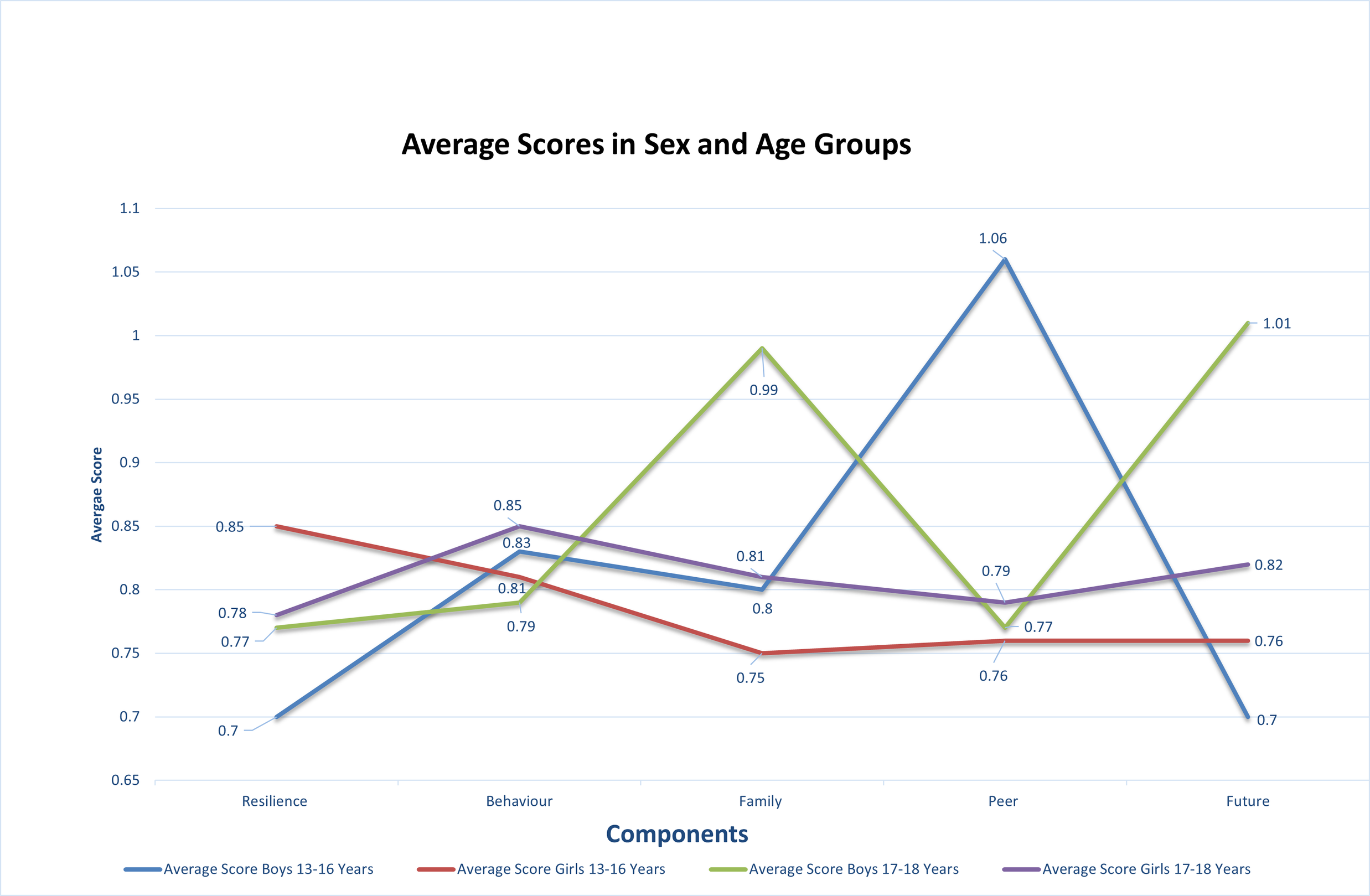

Supplement: S1 Fig — (TIF) [file pone.0295704.s001.tif]
