# Supplementary material for: Poorer subjective mental health among girls: Artefact or real? Examining whether interpretations of what shapes mental health vary by sex
Source: PLoS One. 2023 Dec 27;18(12):e0295704. doi: 10.1371/journal.pone.0295704 (PMC10752563; doi:10.1371/journal.pone.0295704)
Supplement: S1 Table — Stratified by age groups. (DOCX) [file pone.0295704.s002.docx]

**Appendix Table 1_Factor Loadings Analysis of SRH_MH, 5-Items solution. Stratified by age groups**

|  | **13-16 Years** | | | | | **17-18 Years** | | | | |
| --- | --- | --- | --- | --- | --- | --- | --- | --- | --- | --- |
|  | Factor 1  Resilience | Factor 2  Behaviours | Factor 3  Family | Factor 4  Peers | Factor 5 Future | Factor 1  Resilience | Factor 2  Behaviours | Factor 3  Family | Factor 4  Peers | Factor 5 Future |
| Relationship with Parents | .173 | .090 | .827 | -.003 | .095 | .091 | .150 | .698 | -.084 | .115 |
| How treated by parents | .136 | .073 | .826 | .065 | .063 | .119 | .065 | .751 | .003 | .086 |
| Interactions within family | .125 | .105 | .744 | -.011 | .152 | .106 | .036 | .700 | .048 | .134 |
| Family access to money | -.164 | .469 | .468 | .165 | -.039 | -.003 | .136 | .498 | .404 | -.146 |
| Comparing to peers^*^ | .032 | -.128 | -.136 | .693 | .235 | .054 | .093 | -.259 | .579 | .427 |
| Pressure from peers | -.116 | .030 | .130 | .750 | .068 | -.131 | .047 | .057 | .713 | .310 |
| Having a boy/girl friend | .109 | .210 | -.096 | .479 | -.110 | .075 | .161 | -.032 | .573 | -.132 |
| How treated by peers | .084 | .169 | .271 | .548 | .088 | .246 | -.026 | .307 | .469 | .287 |
| Plans for future | .142 | .178 | .056 | .083 | .631 | .186 | .262 | .209 | -.194 | .427 |
| Extracurricular activities^*^ | -.016 | .572 | -.033 | -.174 | .380 | -.090 | .465 | .421 | -.083 | .284 |
| School Pressure | .138 | .030 | .045 | .091 | .693 | .002 | .014 | -.008 | .069 | .779 |
| School performance | .143 | -.016 | .220 | .046 | .738 | .182 | .045 | .126 | .014 | .739 |
| Being part of a community | .180 | .523 | .304 | .171 | -.061 | .059 | .203 | .624 | .120 | -.042 |
| Involvement in community | .029 | .735 | .122 | -.055 | .086 | -.014 | .469 | .502 | .104 | -.061 |
| Self_acceptance | .815 | -.004 | -.057 | -.075 | .101 | .764 | .132 | .023 | .123 | .096 |
| Ability to cope | .788 | -.012 | .127 | .043 | .132 | .783 | .021 | .098 | .023 | .112 |
| Sense of identity | .584 | .261 | .098 | .139 | .060 | .504 | .183 | .434 | -.007 | -.059 |
| Self confidence^*^ | .753 | .049 | .065 | -.046 | .182 | .762 | .321 | -.046 | -.016 | .100 |
| Emotional wellbeing | .775 | .025 | .123 | .011 | -.022 | .742 | .005 | .133 | .094 | .020 |
| A mental health diagnosis | .242 | .445 | .234 | .291 | -.295 | .247 | .027 | .293 | .503 | -.220 |
| Physical wellbeing | .540 | .470 | .249 | .079 | .015 | .389 | .564 | .252 | .183 | .017 |
| Exercise routine^*^ | .175 | .677 | .031 | .004 | .104 | .141 | .761 | .233 | -.068 | .008 |
| Sleeping well | .488 | .334 | .040 | .115 | .237 | .235 | .616 | .025 | .071 | .043 |
| Substance use | -.019 | .498 | .030 | .104 | -.046 | .099 | .375 | .051 | .467 | -.207 |
| Food eaten^*^ | .347 | .457 | .143 | .198 | .114 | .110 | .660 | .171 | .165 | -.009 |
| Screen time | .199 | .390 | -.231 | .128 | .265 | -.059 | .593 | .016 | .229 | .188 |
| Average score with 95%CI (via linear regression) | 0.76  (0.71-0.81) | 0.85  (0.79-0.89) | 0.75  (0.70-0.80) | 0.84  (0.79-0.89) | 0.79  (0.74-0.84) | 0.83  (0.77-0.85) | 0.79  (0.74-0.83) | 0.79  (0.73-0.85) | 0.76  (0.71-0.81) | 0.81  (0.75-0.86) |

KMO measure for sampling adequacy for 13-16 years=0.807; for 17-18 years=0.0800

p. value for Barlett’s test of sphericity for both age groups <0.001

Total percentage of variance explained by five components for 13-16 years=52%; for 17-18 years=53%
